# Supplementary material for: Biomechanical control of vascular morphogenesis by the surrounding stiffness
Source: Nat Commun. 2025 Jul 28;16:6788. doi: 10.1038/s41467-025-61804-z (PMC12304211; doi:10.1038/s41467-025-61804-z)
Supplement: Supplementary file 1 — Supplementary information [file 41467_2025_61804_MOESM1_ESM.pdf]

Supplementary information

Supplementary Figures

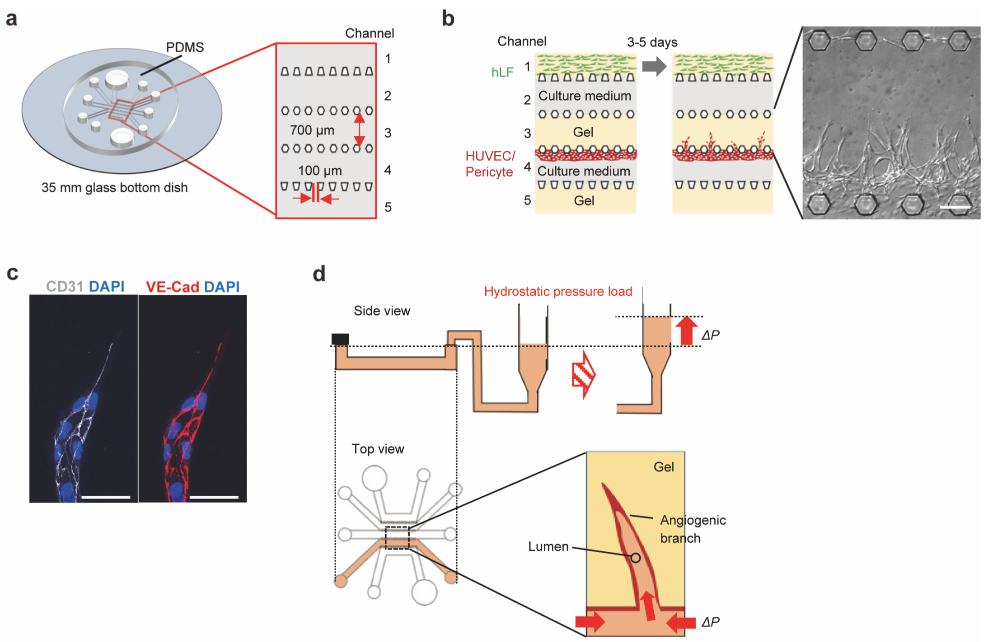

Supplementary Figure 1: An on-chip angiogenesis assay system using ECs and pericytes and a hydrostatic pressure loading system

a, Microfluidic device mounted on a 35 mm glass bottom dish (left). Top view of the area outlined by a red square (right). The device contains five flow channels (1-5), 700 µm in width, each partitioned by microposts (100 µm window). **b**, Schematic illustration of the on-chip angiogenesis assay, in which ECs (HUVECs) were administered via channel 4 with or without pre-administration of pericytes (hPlPCs). Sprouting angiogenesis was induced in fibrin-collagen gel (channel 3) by coculturing human lung fibroblasts (hLFs) in channel 1. A representative DIC image of angiogenic branches 3 days after starting cultivation (right). Scale bars: 100 µm. **c**, Representative confocal z-projection images of on-chip angiogenic branches. Scale bars: 50 µm. **d**, Schematic representation of how to load additional intraluminal pressure (*ΔP*) onto on-chip angiogenic branches by producing hydrostatic pressure differences among culture media. The height of the water surface in a syringe barrel, which had been filled with culture media and connected to channel 4 of the device through a pressure resistant connection tube, was adjusted.

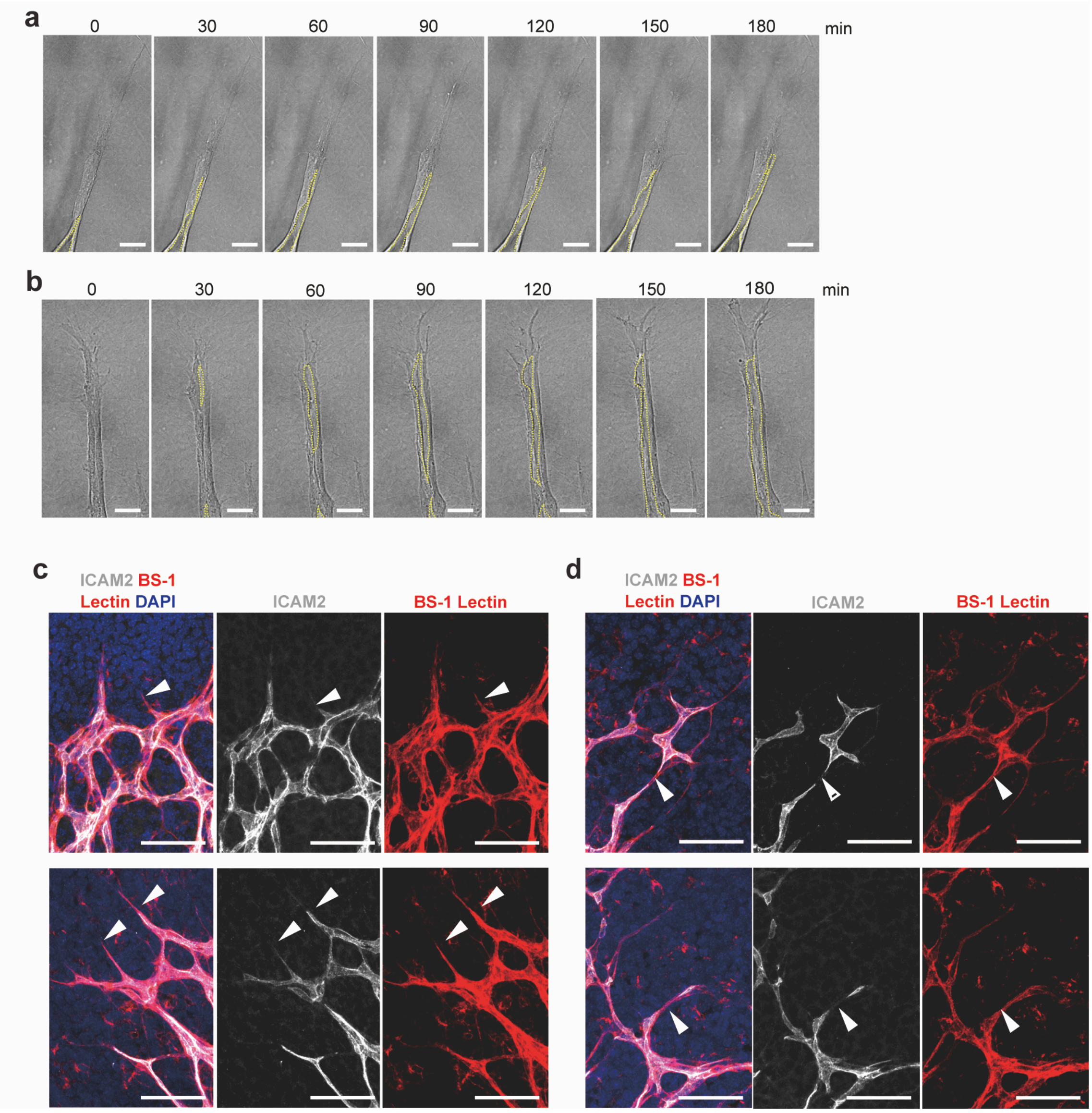

Supplementary Figure 2: Two patterns of *de novo* lumen formation in angiogenesis
a, **b**, Representative time-lapse DIC images showing two different patterns of *de novo* vascular lumen development around the tips of elongating branches in an on-chip angiogenesis assay: Proximal-to-Distal extension (**a**, See also Supplementary Movie 2) and Distal-to-Proximal fusion (**b**, See also Supplementary Movie 3). Yellow lines indicate lumen structures. Scale bars: 25 µm. **c**, **d**, Representative confocal z-projection images of the leading fronts of developing murine retinal vasculature on P3, showing vascular lumen structure. Angiogenic branches at the tip showed a tapered lumen structure toward the distal tip (white arrowheads), possibly corresponding to Proximal-to-Distal extension (**c**). Less frequently, some angiogenic branches at the tip displayed discontinuous structures (white arrowheads) of vascular lumens from the proximal side toward the distal side, possibly suggesting the Distal-to-Proximal fusion pattern of lumen formation seen in the on-chip angiogenesis assay (**d**). Scale bars: 50 µm.

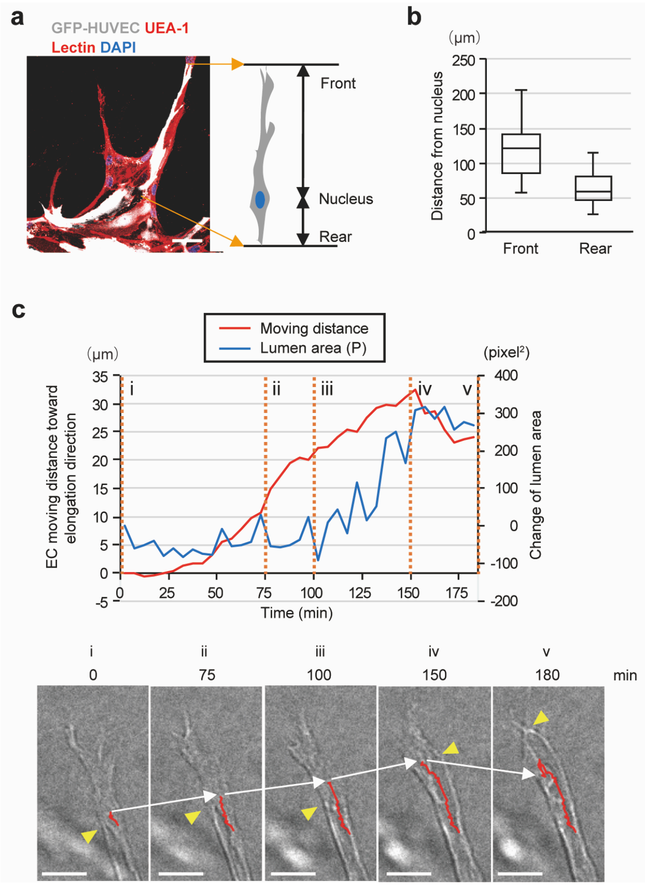

Supplementary Figure 3: Quantification of relationships in dynamics between EC movement and vascular lumen expansion in on-chip angiogenesis

a, **b**, Single EC shape analysis in angiogenic branches using a mosaic method to determine analyzed vascular lumen area. **a**, A representative confocal z-projection image of UEA-1 Lectin positive EC branches including a small population of genetically GFP-labeled HUVECs (GFP-HUVECs) (left). A schematic illustration of a representative cell morphology of GFP-HUVEC moving toward the tip of an angiogenic branch (right). **b**, Box plots of length from the center of the nucleus to the front (205-58 μm) or the rear (166-27 μm) of the GFP-HUVEC (n=40, from 3 independent experiments). Scale bar: 25 μm. All box plots show the interquartile range, with the middle line defining the median, and whiskers show the minimum and maximum values, excluding outliers. Outlier values are defined as being 1.5 times the interquartile range above and below the third and first quartile, respectively. **c**, Kymographs of EC movement (red) and lumen area (proximal area (P) in blue) in elongating branches (top). Serial DIC images of angiogenic branches with trajectory (red line) of the center of the EC nucleus at timepoints indicated in numbers on the kymograph (orange dotted line) (bottom). Tip EC movement decelerated (white arrows from iv to v) when the lumen was developed via Proximal-to-Distal extension (yellow arrowheads) (See also Supplementary Movie 5). Scale bars: 25 μm. Source data are provided as a Source Data file.

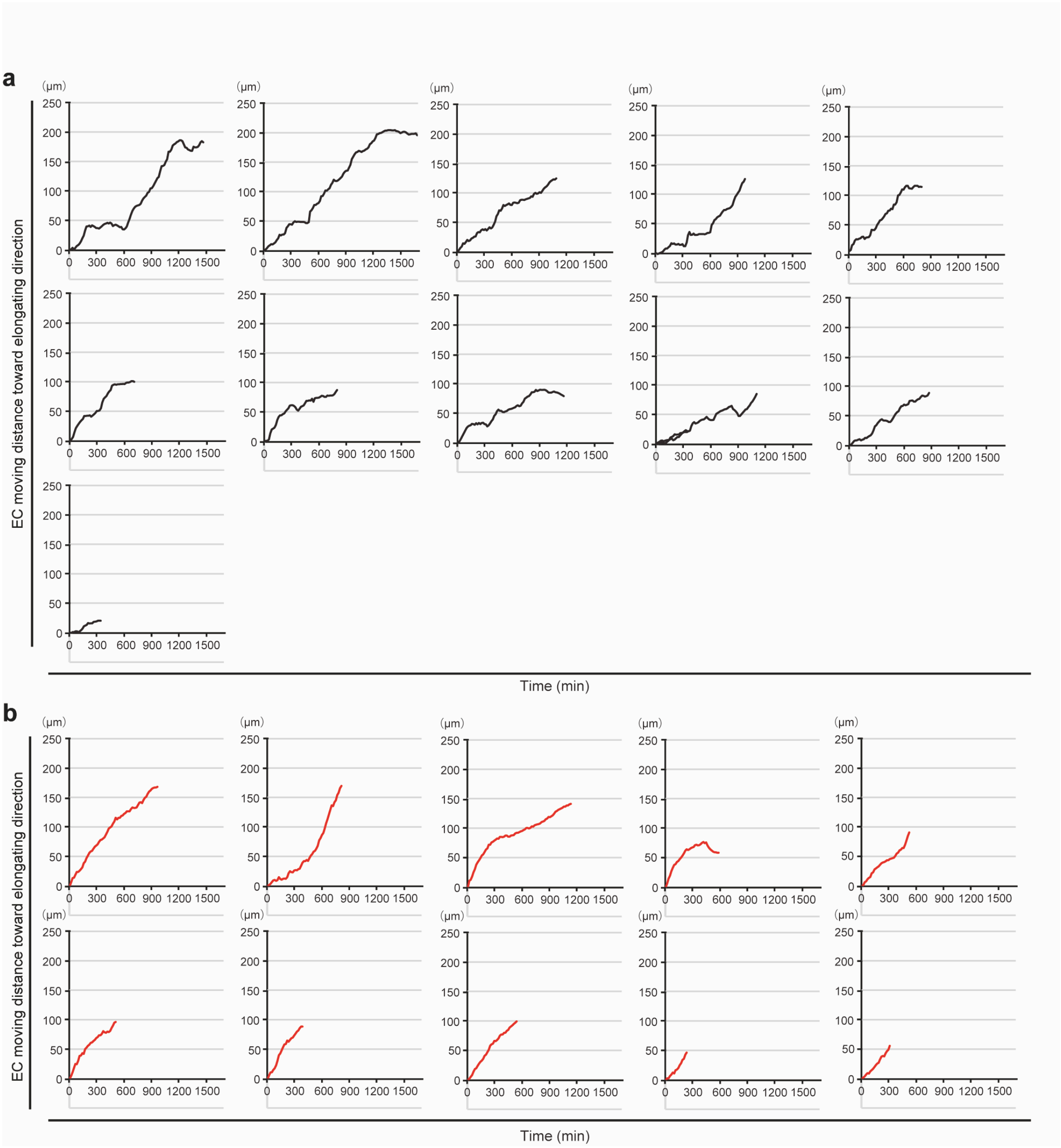

Supplementary Figure 4: Tip EC movement patterns in elongating branches of on-chip angiogenesis using gels without and with TG treatment

Kymographs of individual tip EC movements in elongating angiogenic branches of on-chip angiogenesis by ECs using gel without (**a**) or with (**b**) TG treatment (from 3 independent experiments), which correspond to the data in Fig. 2h. A move forward and stop pattern of tip EC movement was frequently seen in elongating angiogenic branches of on-chip angiogenesis using gels without TG treatment (**a**) but rarely in those using gels with TG treatment (**b**). Source data are provided as a Source Data file.

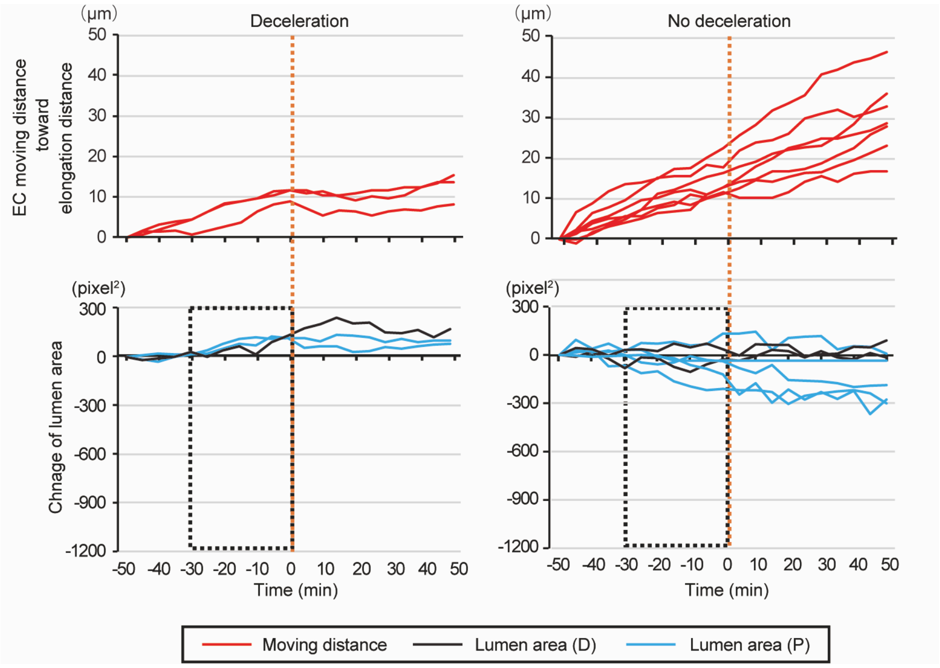

Supplementary Figure 5: Temporal relationship between tip EC movement and lumen expansion in elongating branches of on-chip angiogenesis using gels with TG treatment

Kymographs showing relationships in dynamics between tip EC movement (top) and lumen area (bottom) for 50 minutes before and after the deceleration timepoint (0 minutes, orange dotted line) in the presence (left) and the absence (right) of deceleration of forward tip EC movements (deceleration: n=3; no deceleration: n=7, from 3 independent experiments). For changes of lumen area, data are shown for the predominantly expanded proximal (P) or distal (D) lumen. Dynamics data indicated within squares bounded by black dots were used for analysis of changes in the lumen area for 30 minutes prior to the deceleration timepoint shown in Fig. 1h. Source data are provided as a Source Data file.

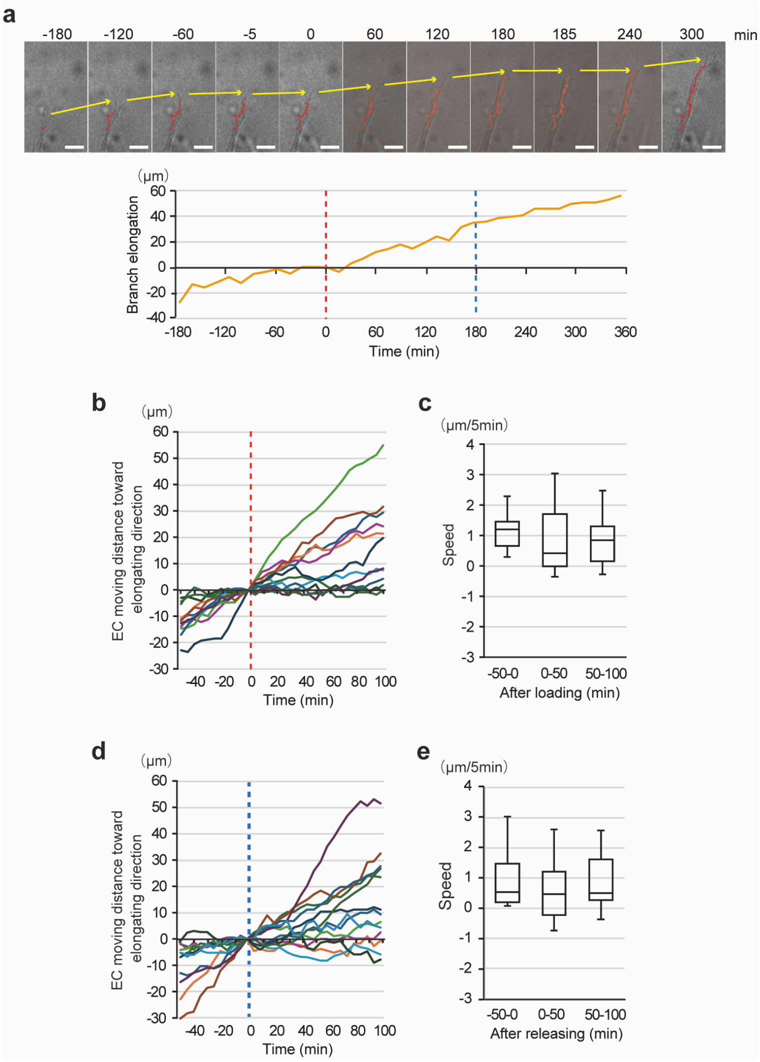

Supplementary Figure 6: Time-lapse imaging of on-chip angiogenesis of ECs as control with no interventions

Physical intervention control experiments for lumen and branch expansions by externally loading intraluminal pressure. **a**, Representative serial DIC images at the elapsed time indicated at the top showing the dynamics in branch elongation, lumen development and tip EC movement in the same timeline, before and after loading and then releasing the additional intraluminal pressure in the intervention group (Fig. 3d) (top, See also Supplementary Movie 9) and kymograph of branch elongation (bottom). In the DIC images, red lines and yellow arrows indicate the trajectory of tip EC displacement and the tip of the branch, respectively. In the kymograph, red and blue dotted lines indicate time points at which additional intraluminal pressure was loaded and released, respectively, in the intervention group. Scale Bars: 20 μm. **b-e,** Quantification of tip EC movement. **b**, Kymograph showing the dynamics in tip EC movement in the same timeline, 50 minutes before and 100 minutes after loading additional intraluminal pressure in the intervention group (time=0, dotted red line). **c**, Box plots of averaged tip EC movement speeds in the same timeline, 50 minutes before, 50 minutes and 50-100 minutes after the pressure load in the intervention group (each group: n=12 from 3

independent experiments). **d**, Kymograph showing the dynamics in tip EC movement in the same timeline, 50 minutes before and 100 minutes after the release of additional intraluminal pressure in the intervention group (time=0, dotted red line). **e**, Box plots of averaged tip EC movement speeds in the same timeline, 50 minutes before, 50 minutes and 50-100 minutes after pressure release in the intervention group (each group: n=13, from 3 independent experiments). All box plots show the interquartile range, with the middle line defining the median, and whiskers show the minimum and maximum values, excluding outliers. Outlier values are defined as being 1.5 times the interquartile range above and below the third and first quartile, respectively. Wilcoxon signed rank test with a Bonferroni correction (**c**, **e**). Source data are provided as a Source Data file.

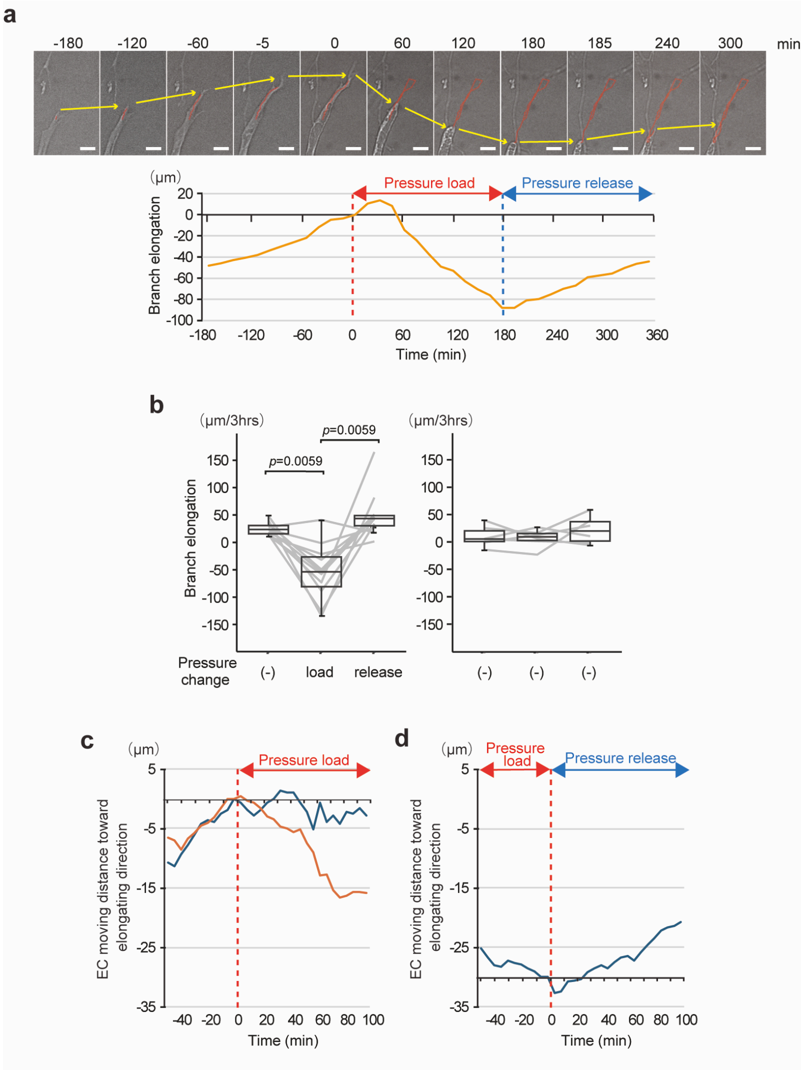

Supplementary Figure 7: Time-lapse imaging of on-chip angiogenesis of ECs at the 2nd intervention cycle and the corresponding analyses

The second cycle of physical intervention for lumen and branch expansions by externally loading intraluminal pressure. **a**, Representative serial DIC images at the elapsed time indicated at the top showing the dynamics in branch elongation, lumen development and tip EC movement before and after loading and then releasing additional intraluminal pressure (top, See also Supplementary Movie 10) and kymograph of branch elongation (bottom). In the DIC images, red lines and yellow arrows indicate the trajectory of tip EC displacement and the tip of the branch, respectively. In the kymograph, red and blue dotted lines indicate time points at which additional intraluminal pressure was loaded and released, respectively. Scale Bars: 20 µm. **b**, Box plots of averaged branch elongation 3 hours before (pressure change (-)) and after (load) the 2nd pressure loading and 3 hours after the release (release), merged with individual plots showing the branch elongation changes in response to loading and then the release of intraluminal pressure (pressure load and release: n=11, from 3 independent experiment; control: n=6, from 2 independent experiments). All box plots show the interquartile range, with the middle line defining the median, and whiskers show the minimum and maximum values, excluding

outliers. Outlier values are defined as being 1.5 times the interquartile range above and below the third and first quartile, respectively. **c**, Kymograph showing the dynamics in tip EC movement 50 minutes before and 100 minutes after loading additional intraluminal pressure (time=0, dotted red line). **d**, Kymograph showing the dynamics in tip EC movement 50 minutes before and 100 minutes after the release of additional intraluminal pressure (time=0, dotted red line). Wilcoxon signed rank test with a Bonferroni correction (**b**). Source data are provided as a Source Data file.

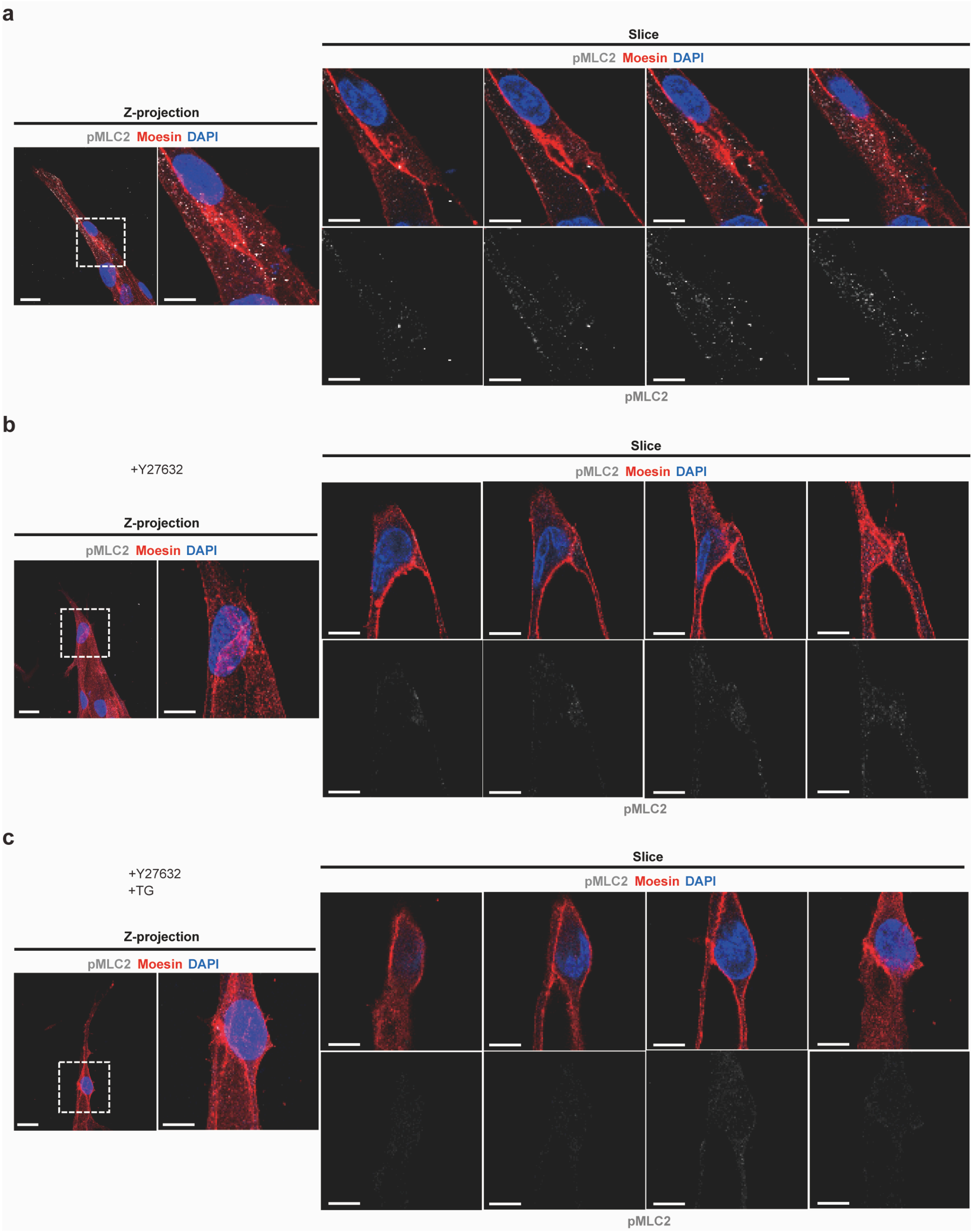

Supplementary Figure 8: Phosphorylated myosin light chain 2 localization in the branch around forming *de novo* lumen in on-chip angiogenesis without and with ROCK inhibitor and without and with TG treatment

Representative confocal z-projection images of on-chip angiogenesis, magnified images of the areas enclosed within squares bounded by white dots in the left images (z-

projection), and their corresponding confocal *x-y* slice images at specific z-positions (slice) of the non-treated group (**a**), ROCK inhibitor (Y27632) -treated group (**b**) and both ROCK inhibitor and TG-treated groups (**c**). Scale bars: 10 μm for confocal z-projection images in the right and *x-y* slice images and 20 μm for confocal z-projection images in the left.

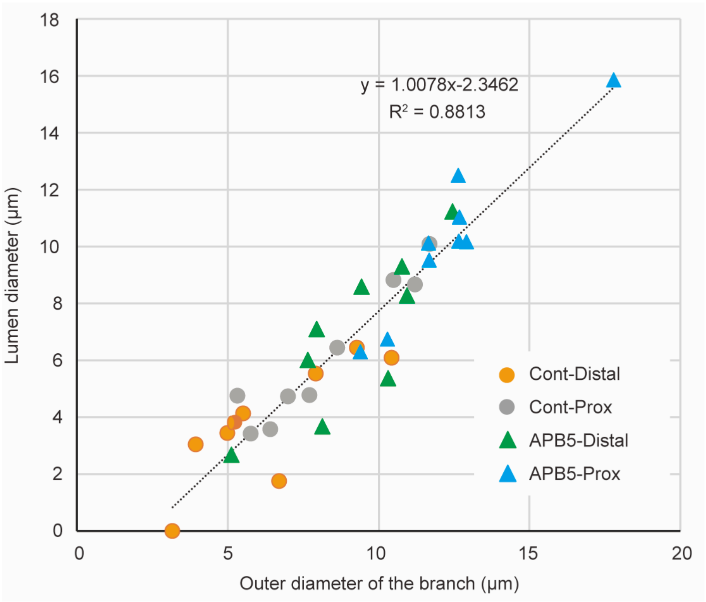

Supplementary Figure 9: Diameter size correlation between lumen and branch in murine retinal angiogenic branches

A dot graph showing relationship between lumen diameter and branch diameter sizes in P4 murine retinal branches. The data were obtained from portions around the tip (Distal) and more proximal sites (Prox) in murine retinal angiogenic branches with (Cont) and without (APB5) pericyte coverage (Cont-Distal, n=9, Cont-Prox, n=9; APB5-Distal, n=9, APB5-Prox, n=9, from 2 independent experiments). Source data are provided as a Source Data file.

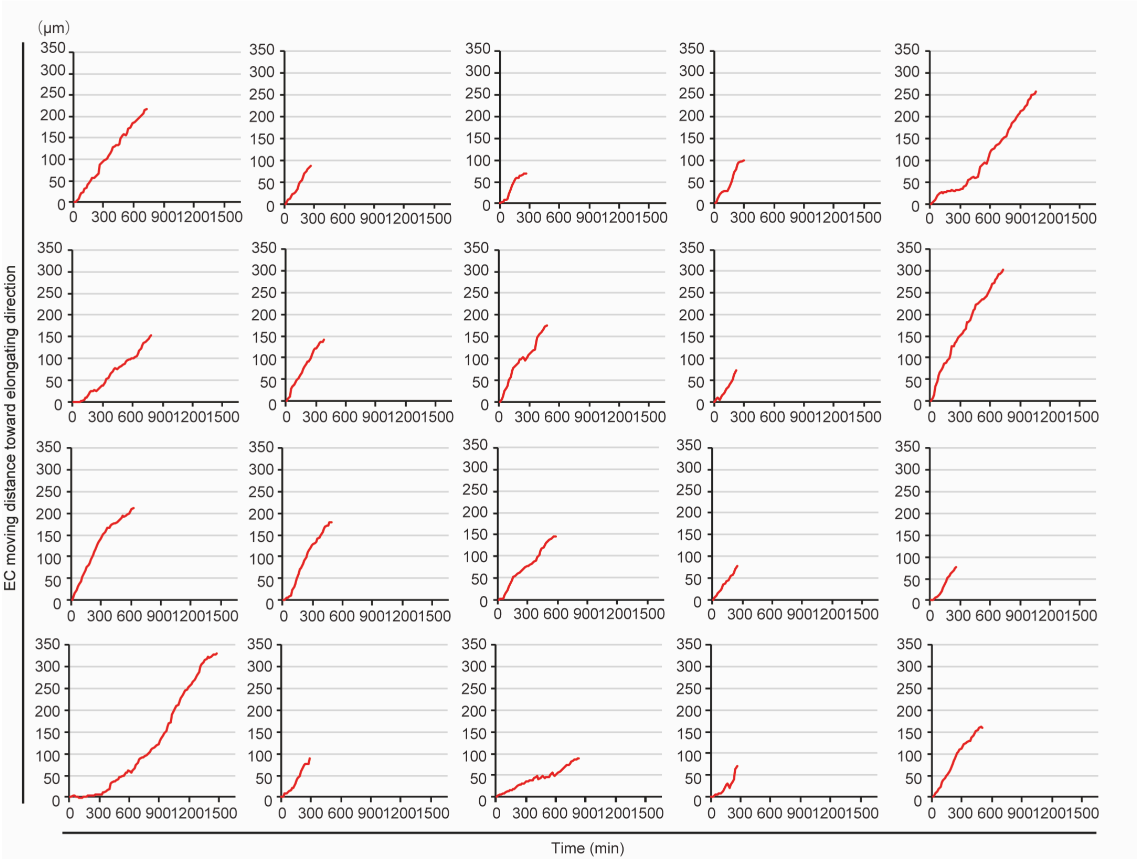

Supplementary Figure 10: Tip EC movement patterns in elongating branches of on-chip angiogenesis with pericyte coculture

Kymographs of individual tip EC movements in elongating angiogenic branches during coculture with pericytes, which correspond to the data in Fig. 5a (from 3 independent experiments). Source data are provided as a Source Data file.

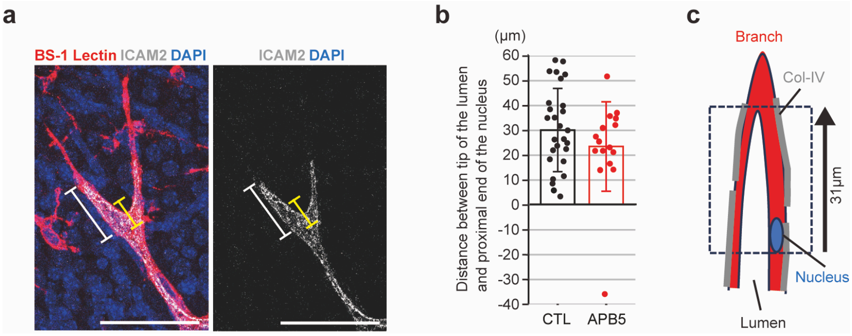

Supplementary Figure 11: Setting of area analyzed for quantification of Col-IV or laminin deposition on VBM in murine retinal angiogenic branches

a, Representative confocal z-projection images of leading front of murine retinal angiogenic branches on P4. **b**, Distances from the distal tip of the lumen to the proximal ends of the nuclei of the tip ECs on confocal z-projection images were measured in murine retinal angiogenic branches with (APB) and without (CTL) anti-PDGFRβ antibody treatment (CTL: n=28; APB5: n=19, from 2 independent experiments). **c**, Schematic representation of VBM area analyzed for quantification of Col-IV or laminin deposition. Based on the analyzed data shown in **b**, the perivascular area was set within 31 μm distal from the proximal ends of the nuclei of the tip ECs. Data are expressed as means ± SD. Scale bars: 25 μm. Source data are provided as a Source Data file.

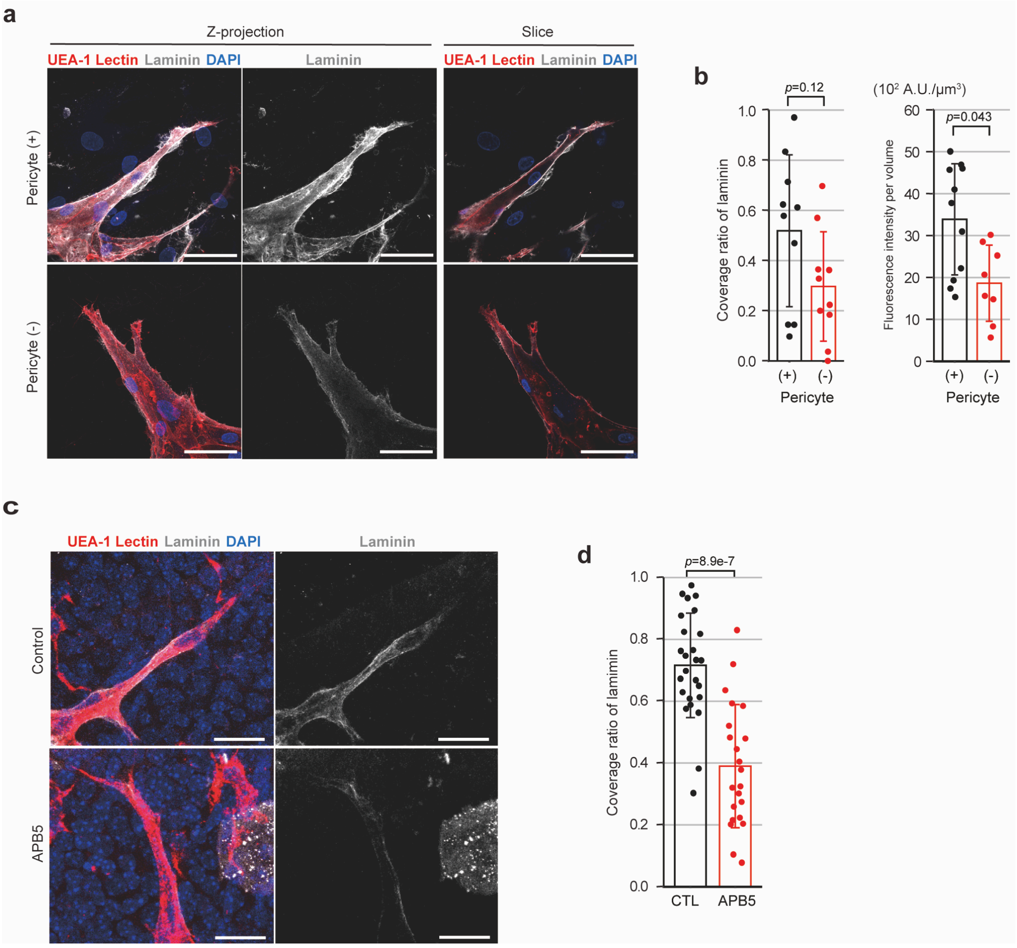

Supplementary Figure 12: Laminin deposition on VBM around the tip at the site of *de novo* vascular lumen formation in on-chip angiogenesis and in murine retinal angiogenesis

a, Representative confocal z-projection images (z-projection) and confocal *x-y* slice images at specific z-positions (slice) of on-chip angiogenic branches with and without pericyte coculture. **b**, Quantitative analysis of laminin deposition on VBM, evaluated by the following parameters: Coverage ratio (left, pericyte (-): n=10; pericyte (+): n=10, from 3 independent experiments) and Fluorescence intensity per volume (right, pericyte (-): n=9; pericyte (+): n=10, from 3 independent experiments). **c**, Representative confocal z-projection images of murine retinal angiogenic branches on P4, with and without anti-PDGFRβ neutralizing antibody (APB5) treatment that induces removal of pericyte coverage. **d**, Quantitative analysis of laminin deposition on VBM in the perivascular area around the site of *de novo* lumen formation (control (CTL): n=25; APB5: n=22, from 2 independent experiments). Data are expressed as means ± SD. Scale bars: 20 μm (**d**) and 50 μm (**a**). Two-sided Mann-Whitney U test. Source data are provided as a Source Data file.

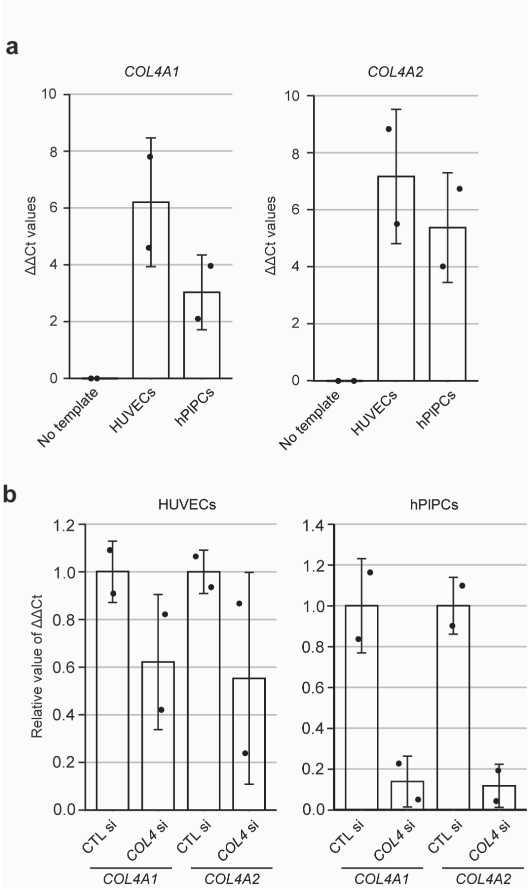

Supplementary Figure 13: Expressions of *COL4A1* and *COL4A2* genes and their knock-down using siRNAs in ECs and pericytes

a, Quantitative PCR data for gene expressions of *COL4A1* and *COL4A2* in cultured HUVECs and hPlPCs (pericytes). Data are expressed as means of ΔΔCT values (n=3 for each group, from 2 independent experiments). **b**, Quantitative PCR data for gene expressions of *COL4A1* and *COL4A2* in HUVECs and hPlPCs, treated with *COL4A* si (siRNAs for both *COL4A1* and *COL4A2* genes) or control (CTL) si for 2 days. Data are expressed as the means of the relative values of ΔΔCT to one in CTL si (n=2 for each group, from 2 independent experiments). Source data are provided as a Source Data file.

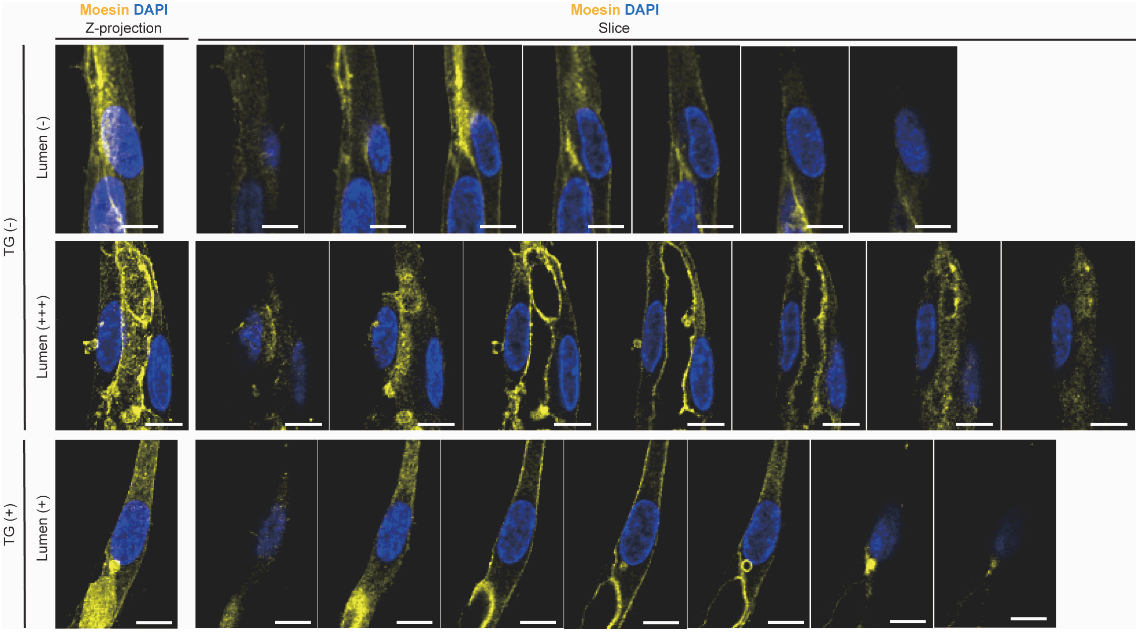

Supplementary Figure 14: Patterns of lumen development around the tip ECs in on-chip angiogenesis by ECs using gels without and with TG treatment

Representative confocal z-projection images and their corresponding confocal *x-y* slice images at specific z-positions (slice), showing different degrees of lumen development around the tip ECs (well developed: lumen (+++); less developed: lumen (+); not developed: lumen (-)). Scale bars: 10 µm.

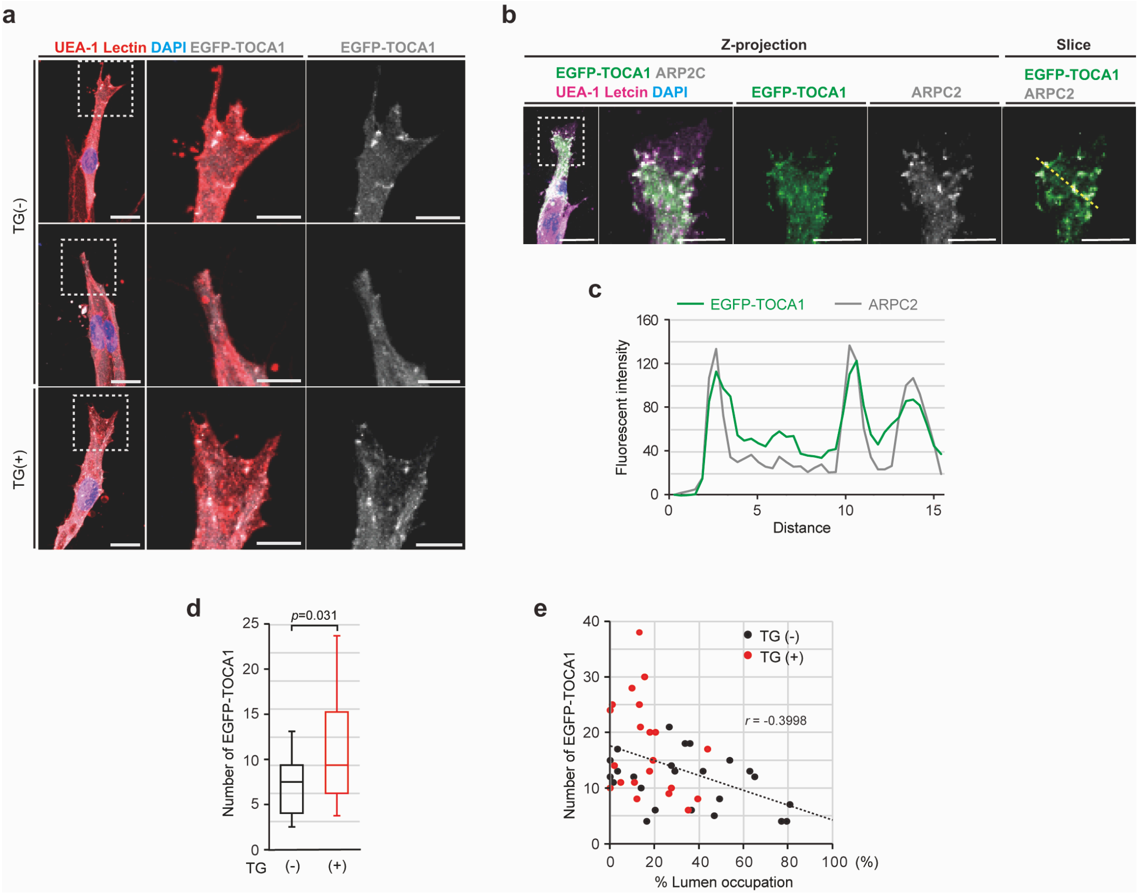

Supplementary Figure 15: Relationship between EGFP-TOCA1 localization patterns around the tips of branches and nearby lumen development in on-chip angiogenesis without and with TG treatment of the gel

a, Representative z-projection confocal images, showing different localization patterns of EGFP-TOCA1 around the leading edges of angiogenic branches using gels without (TG (-), top and middle) and with (TG (+), bottom) TG treatment. Areas surrounded by squares with a boundary of white dots are shown magnified on the right. **b**, **c**, Line scan profiles of fluorescent intensity of EGFP-TOCA1 and ARPC2 along the yellow dotted line indicated in the confocal *x-y* slice image of the left panel of **b** (right), showing colocalization of EGFP-TOCA1 with Arp2/3 complexes (**c**). **d**, Quantification of EGFP-TOCA1 localization around the leading edges of angiogenic branches (TG (-): n=25 branches; TG (+): n=21 branches, from 3 independent experiments). All box plots show the interquartile range, with the middle line defining the median, and whiskers show the minimum and maximum values, excluding outliers. Outlier values are defined as being 1.5 times the interquartile range above and below the third and first quartile, respectively. **e**, Distribution plot showing relationship between EGFP-TOCA1 localization around the leading edge of the branch and lumen development without (black circles) and with (red circles) TG treatment of the gel (TG (-): n=25 branches; TG (+): n=21 branches, from 3 independent experiments). Scale bars: 10 μm (right panels of **a**, **b**) and 20 μm (left panels of **a**, **b**). Two-sided Mann-Whitney U test (**d**). Source data are provided as a Source Data file.

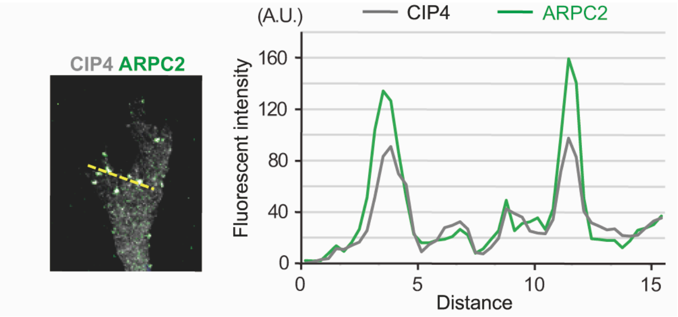

Supplementary Figure 16: Colocalization of CIP4 with Arp2/3 complexes at the leading edge of on-chip angiogenic branch

Line scan profiles of fluorescent intensities of CIP4 and ARPC2 along the yellow dotted line indicated in the confocal *x-y* slice image (left), showing colocalization of CIP4 with Arp2/3 complexes (right). Source data are provided as a Source Data file.

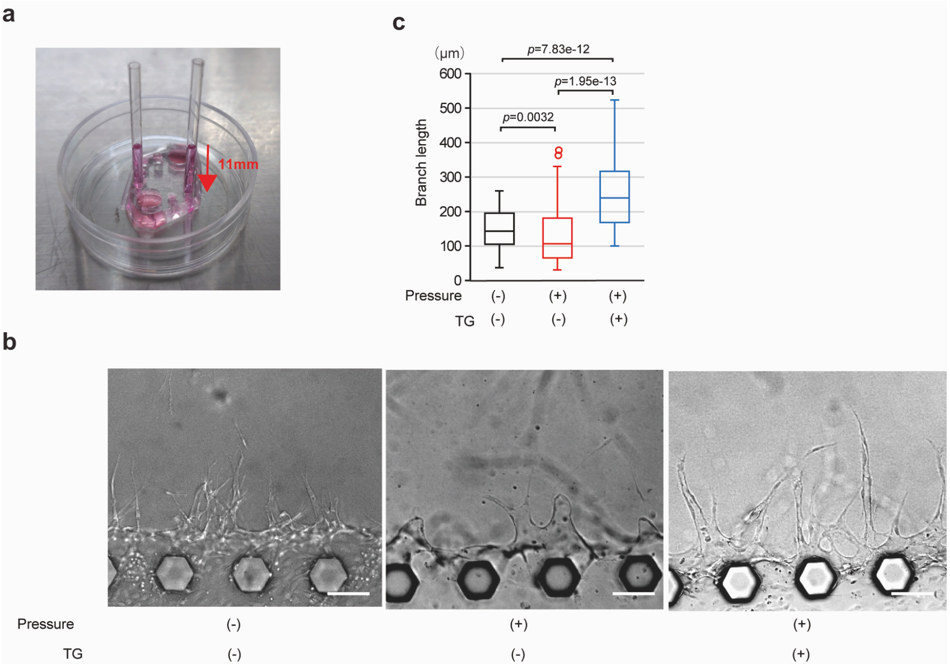

Supplementary Figure 17: Effects of intraluminal pressure load on branch elongation in on-chip angiogenesis with and without TG treatment of the gel
a, Intraluminal pressure loading into luminal space of the angiogenic branches by placing capillaries filled with culture media (11 mm in height). **b**, **c**, Changes in branch elongation of on-chip angiogenesis without or with TG treatment of the gel by intraluminal pressure loading. **b**, Representative DIC images. Scale bars: 100 µm. **c**, Quantification of branch length (Pressure (P) (-)/TG (-): n=90 branches; P (+)/TG (-): n=82 branches; P (+)/TG (+): n=94 branches, from 3 independent experiments). All box plots show the interquartile range, with the middle line defining the median, and whiskers show the minimum and maximum values, excluding outliers. Outlier values are defined as being 1.5 times the interquartile range above and below the third and first quartile, respectively. Mann-Whitney U test with a Bonferroni correction. Source data are provided as a Source Data file.

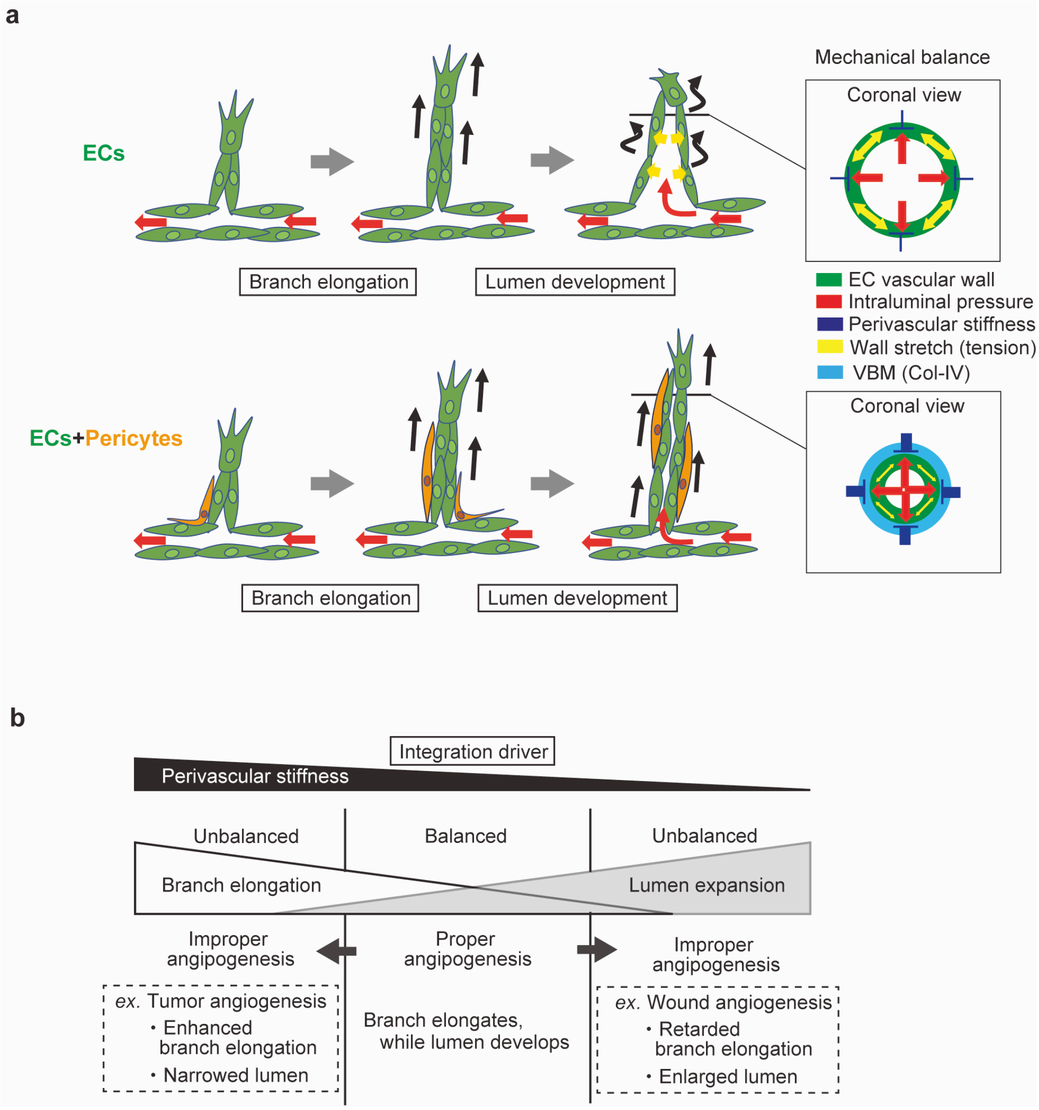

Supplementary Figure 18: Schematic representations of proposed models of a mechanism integrating branch elongation and lumen expansion in angiogenic morphogenesis and the involvement of their breakdown in improper angiogenesis

a, A biomechanical control model of angiogenic morphogenesis. Upper panel, forward directional movement (black arrows) of ECs, including tip ECs, drives efficient branch elongation. Immediately after the vascular lumen emerges in the elongating branch, blood flows into the lumen, driven by cardiac pumping pressure (blood flow, red arrows), which increases intraluminal pressure followed by circumferential expansion of both the lumen and the branch. The expansion of the lumen and the branch slows forward movement of ECs (winding black arrows). Lower panel, in physiological angiogenesis during the developmental stage, perivascular pericytes adjacent to ECs forming the branch counter-regulate the blood flow-induced inhibition of EC forward movement and branch elongation. Excessive circumferential expansion of the lumen and branch is biomechanically prevented by pericyte-dependent stiffening of the perivascular environment due to enhanced deposition of Col-IV on VBM. In this model, the physical property of the perivascular environment serves as the main driver integrating branch

elongation and lumen development by controlling the mechanical balance among factors governing perivascular stiffness, intraluminal pressure and vascular wall stretch (tension). **b**, A mechanistic model centrally explains proper and improper angiogenesis in different types of situations. In physical situations like the developmental stage, angiogenesis proceeds properly, with branch elongation, while the vascular lumen develops concurrently, under an appropriate mechanical balance among factors governing perivascular stiffness, intraluminal pressure and vascular wall (stretch) tension (Balanced). However, in certain pathological situations, this mechanical balance can be distorted (Unbalanced): for instance, in wound angiogenesis, ectopically increased intraluminal pressure can result in an unbalanced mechanical state, which hinders branch elongation and enlarges both the lumen and the branch. In contrast, in tumor angiogenesis, the perivascular tumor micro-environment is stiffened and perivascular interstitial pressure is elevated, which can induce an unbalanced mechanical state, resulting in enhanced branch elongation with a narrow lumen and thin branch.

Supplemental Tables

Supplementary Table 1. Primer sequences for qPCR

**qPCR**		
COL4A1	CCAGGGGTCGGAGAGAAAG	GGTCCTGTGCCTATAACAATTCC
COL4A2	TTATGCACTGCCTAAAGAGGAGC	CCCTTAACTCCGTAGAAACCAAG
GAPDH	GTCTCCTCTGACTTCAACAGCG	ACCACCCTGTTGCTGTAGCCAA
