# Supplementary material for: Biomechanical control of vascular morphogenesis by the surrounding stiffness
Source: Nat Commun. 2025 Jul 28;16:6788. doi: 10.1038/s41467-025-61804-z (PMC12304211; doi:10.1038/s41467-025-61804-z)
Supplement: Supplementary file 2 — Description of Additional Supplementary Files [file 41467_2025_61804_MOESM2_ESM.pdf]

Description of Additional Supplementary Files

File name: Supplementary Movie 1
Description: Time-lapse imaging of on-chip angiogenesis of ECs. Images were obtained every 15 min. Elapsed time (hr:min) is shown in the upper left corner. See also Fig. 1a.

File name: Supplementary Movie 2
Description: Time-lapse imaging of on-chip angiogenesis of ECs, where the lumen developed toward the distal tip (Proximal-to-Distal extension). Images were obtained every 2 min. Elapsed time (hr:min) is shown in the upper left corner. See also Supplementary Fig. 2a.

File name: Supplementary Movie 3
Description: Time-lapse imaging of on-chip angiogenesis of ECs, where a large vacuole like structure initially emerged around the tip of the branch and it connected to the pre-existing lumen (Distal-to-Proximal fusion). Images were obtained every 2 min. Elapsed time (hr:min) is shown in the upper left corner. See also Supplementary Fig. 2b.

File name: Supplementary Movie 4
Description: : Time-lapse imaging of on-chip angiogenesis of ECs, where tip EC decelerated immediately after the lumen development by Distal-to-Proximal fusion. Red line indicates the trajectory of the tip EC. Images were obtained every 5 min. Elapsed time (hr:min) is shown in the upper left corner. See also Fig. 1f.

File name: Supplementary Movie 5
Description: Time-lapse imaging of on-chip angiogenesis of ECs, where tip EC decelerated when the lumen was developed by Proximal-to-Distal extension. Red line indicates the trajectory of the tip EC. Images were obtained every 5 min. Elapsed time (hr:min) is shown in the upper left corner. See also Supplementary Fig. 3c.

File name: Supplementary Movie 6
Description: Time-lapse imaging of on-chip angiogenesis of ECs, where tip EC maintained forward movement without deceleration, while the lumen did not expand. Red line indicates the trajectory of the tip EC. Images were obtained every 5 min. Elapsed time (hr:min) is shown in the upper left corner. See also Fig. 1g

File name: Supplementary Movie 7

Description: Time-lapse imaging of on-chip angiogenesis of ECs in the ECM treated with TG. Tip EC continued forward movement, while the size of nearby lumen remained almost constant. Red line indicates the trajectory of the tip EC. Images were obtained every 5 min. Elapsed time (hr:min) is shown at the top of the movie. See also Fig. 2j.

File name: Supplementary Movie 8
Description: Time-lapse imaging of on-chip angiogenesis of ECs with an ectopic load-release cycle of intraluminal pressure. The movie shows from 3 hr before the pressure load to 3 hr after the pressure release. Red line indicates the trajectory of the tip of the branch. Images were obtained every 5 min. Elapsed time (min) is shown in the upper left corner and indicates the time before or after the pressure load started. See also Fig. 3d.

File name: Supplementary Movie 9
Description: : Time-lapse imaging of on-chip angiogenesis of ECs without changes of intraluminal pressure. Red line indicates the trajectory of the tip of the branch. Images were obtained every 5 min. Elapsed time (min) is shown in the upper left corner and indicates the time before or after the pressure load started in the intervention group. See also Supplementary Fig. 6.

File name: Supplementary Movie 10
Description: Time-lapse imaging of on-chip angiogenesis of ECs with the second load-release cycle of intraluminal pressure. The movie shows from 3 hr before the pressure load to 3 hr after the pressure release. Red line indicates the trajectory of the tip of the branch. Images were obtained every 5 min. Elapsed time (min) is shown in the upper left corner and indicates the time before or after the second pressure load started. See also Supplementary Fig. 7.

File name: Supplementary Movie 11
Description: Time-lapse imaging of on-chip angiogenesis of ECs with pericyte coculture. Red line indicates the trajectory of the tip EC. Images were obtained every 5 min. Elapsed time (hr:min) is shown in the upper left corner. See also Fig. 5c.
