# Supplementary material for: Biomechanical control of vascular morphogenesis by the surrounding stiffness
Source: Nat Commun. 2025 Jul 28;16:6788. doi: 10.1038/s41467-025-61804-z (PMC12304211; doi:10.1038/s41467-025-61804-z)
Supplement: Supplementary file 14 — Reporting Summary [file 41467_2025_61804_MOESM14_ESM.pdf]

nature portfolio

Corresponding author(s): NCOMMS-24-10909B

Last updated by author(s): Jun 18, 2025

# Reporting Summary

Nature Portfolio wishes to improve the reproducibility of the work that we publish. This form provides structure for consistency and transparency in reporting. For further information on Nature Portfolio policies, see our Editorial Policies and the Editorial Policy Checklist.

## Statistics

For all statistical analyses, confirm that the following items are present in the figure legend, table legend, main text, or Methods section.

| n/a | Confirmed | |
|---|---|---|
| [ ] | [x] | The exact sample size (*n*) for each experimental group/condition, given as a discrete number and unit of measurement |
| [ ] | [x] | A statement on whether measurements were taken from distinct samples or whether the same sample was measured repeatedly |
| [ ] | [x] | The statistical test(s) used AND whether they are one- or two-sided<br>*Only common tests should be described solely by name; describe more complex techniques in the Methods section.* |
| [x] | [ ] | A description of all covariates tested |
| [ ] | [x] | A description of any assumptions or corrections, such as tests of normality and adjustment for multiple comparisons |
| [ ] | [x] | A full description of the statistical parameters including central tendency (e.g. means) or other basic estimates (e.g. regression coefficient) AND variation (e.g. standard deviation) or associated estimates of uncertainty (e.g. confidence intervals) |
| [ ] | [x] | For null hypothesis testing, the test statistic (e.g. *F*, *t*, *r*) with confidence intervals, effect sizes, degrees of freedom and *P* value noted<br>*Give P values as exact values whenever suitable.* |
| [x] | [ ] | For Bayesian analysis, information on the choice of priors and Markov chain Monte Carlo settings |
| [x] | [ ] | For hierarchical and complex designs, identification of the appropriate level for tests and full reporting of outcomes |
| [ ] | [x] | Estimates of effect sizes (e.g. Cohen's *d*, Pearson's *r*), indicating how they were calculated |

*Our web collection on statistics for biologists contains articles on many of the points above.*

## Software and code

Policy information about availability of computer code

Data collection

Image acquisition for murine retinal samples: Fluorescence images were acquired using a fluorescent microscope (BZ-X810, Keyence). All confocal images were obtained using a confocal laser-scanning microscope (FluoView FV1200, Olympus), equipped with a GaAsP detector (Olympus) operated by FV10-ASM4.2 software (Olympus) and using a confocal laser-scanning microscope (TCS-SP8, Leica), equipped with a Hybrid Detector (Leica HyD, Leica), operated by LASX version2.0.1.14392 (Leica).

Image acquisition for on-chip angiogenesis assays: All DIC and fluorescent images were obtained by a commercially available fluorescent inverted microscope (IX83, Olympus), equipped with a CMOS camera (ORCA-Flash4.0, Hamamatsu Photonics) operated by MetaMorph operating software version 7.10.1.161 (Molecular device). All confocal images were obtained using a confocal laser-scanning microscope (FluoView FV1200, Olympus), equipped with a GaAsP detector (Olympus) operated by FV10-ASM4.2 software (Olympus) and using a confocal laser-scanning microscope (TCS-SP8, Leica), equipped with a Hybrid Detector (Leica HyD, Leica), operated by LASX version2.0.1.14392 (Leica).

Data collection for quantitative PCR analysis: Applied Biosystems StepOnePlus (Applied Biosystems).

| Data analysis | Analysis for imaging data: All image data were processed using Fiji/ImageJ 2.1.0/1.54b for analyses and presentation, as mentioned in Methods. After appropriate image processing, all images were subsequently analyzed for quantification using the Fiji/ImageJ 2.1.0/1.54b (http://fiji.sc.) and Java (v 17.0.1), as mentioned in Methods.<br><br>Statistical analysis: Statistical analyses for all data were carried out using R 4.2.0. |
|---|---|

For manuscripts utilizing custom algorithms or software that are central to the research but not yet described in published literature, software must be made available to editors and reviewers. We strongly encourage code deposition in a community repository (e.g. GitHub). See the Nature Portfolio guidelines for submitting code & software for further information.

## Data

Policy information about availability of data

All manuscripts must include a data availability statement. This statement should provide the following information, where applicable:

- Accession codes, unique identifiers, or web links for publicly available datasets
- A description of any restrictions on data availability
- For clinical datasets or third party data, please ensure that the statement adheres to our policy

The authors declare that all data supporting the findings of this study are available within the main text and supplementary materials. Raw data to generate all graphs within the Figures and Supplementary Figures in the Supplementary Figures are provided as a Source Data File. Source data are provided with this paper.

## Research involving human participants, their data, or biological material

Policy information about studies with human participants or human data. See also policy information about sex, gender (identity/presentation), and sexual orientation and race, ethnicity and racism.

| Reporting on sex and gender | This study include no research involving human participants, their data, or biological material. |
|---|---|
| Reporting on race, ethnicity, or other socially relevant groupings | *Please specify the socially constructed or socially relevant categorization variable(s) used in your manuscript and explain why they were used. Please note that such variables should not be used as proxies for other socially constructed/relevant variables (for example, race or ethnicity should not be used as a proxy for socioeconomic status).*<br>*Provide clear definitions of the relevant terms used, how they were provided (by the participants/respondents, the researchers, or third parties), and the method(s) used to classify people into the different categories (e.g. self-report, census or administrative data, social media data, etc.)*<br>*Please provide details about how you controlled for confounding variables in your analyses.* |
| Population characteristics | *Describe the covariate-relevant population characteristics of the human research participants (e.g. age, genotypic information, past and current diagnosis and treatment categories). If you filled out the behavioural & social sciences study design questions and have nothing to add here, write "See above."* |
| Recruitment | *Describe how participants were recruited. Outline any potential self-selection bias or other biases that may be present and how these are likely to impact results.* |
| Ethics oversight | *Identify the organization(s) that approved the study protocol.* |

Note that full information on the approval of the study protocol must also be provided in the manuscript.

# Field-specific reporting

Please select the one below that is the best fit for your research. If you are not sure, read the appropriate sections before making your selection.

[x] Life sciences    [ ] Behavioural & social sciences    [ ] Ecological, evolutionary & environmental sciences

For a reference copy of the document with all sections, see nature.com/documents/nr-reporting-summary-flat.pdf

# Life sciences study design

All studies must disclose on these points even when the disclosure is negative.

| Sample size | No statistical method was used to predetermined the sample size for all experiments. Sample size for individual quantification experiment was determined by analyzing all branches or selected branches in each experiment through at least 3 independent experiments except for a part of experiment for the Supplementary Figure to sufficiently secure data reproducibility as well as to prevent biased sampling.<br><br>Exact sample sizes are indicated in the figure legends. |
|---|---|
| Data exclusions | No data were excluded. |
| Replication | All experiments, except for a part of experiments for the Supplementary Figure, were repeated at least 3 times to secure reproducibility. All |

replicated experiments were successful.

Randomization

In on-chip angiogenesis assays, all cultured cells were randomly allocated into different experimental groups in individual assays. For quantification analysis, to limit bias, data were collected from all branches or cells observed in each chip or randomly selected branches in each chip.

For murine retinal analysis, mice or eyes analyzed were randomly selected for each experimental group.

Blinding

Blinding during data collection was not performed since the same investigator did all experimental processes from group allocation to data analysis.

# Reporting for specific materials, systems and methods

We require information from authors about some types of materials, experimental systems and methods used in many studies. Here, indicate whether each material, system or method listed is relevant to your study. If you are not sure if a list item applies to your research, read the appropriate section before selecting a response.

## Materials & experimental systems

| n/a | Involved in the study |
|---|---|
| ☐ | ☒ Antibodies |
| ☐ | ☒ Eukaryotic cell lines |
| ☒ | ☐ Palaeontology and archaeology |
| ☐ | ☒ Animals and other organisms |
| ☒ | ☐ Clinical data |
| ☒ | ☐ Dual use research of concern |
| ☒ | ☐ Plants |

## Methods

| n/a | Involved in the study |
|---|---|
| ☒ | ☐ ChIP-seq |
| ☒ | ☐ Flow cytometry |
| ☒ | ☐ MRI-based neuroimaging |

## Antibodies

Antibodies used

Immunofluorescence staining - Primary: rabbit anti-VE-cadherin (D87F2) mAb (Cell Signaling, 2500), mouse anti-CD31 (WM59) mAb (BioLegend, 303102), rabbit anti-GOLPH4 mAb (Abcam, ab28049), goat anti-ARPC2 pAb (Novus Biologicals, NB100-1037), rabbit anti-TRIP10 pAb (Proteintech, 10798-1-AP), rabbit anti-human PDGFRβ (Y92) mAb (OriGene, TA300416) , goat anti-collagen type IV Ab (SouthernBiotech, 1340-01), rabbit anti-laminin pAb(Abcam, ab11575), rabbit anti-NG2 pAb(Millipore, AB5320), rat anti-mouse CD31 (MEC13.3) mAb (BD Pharmingen, 550274), rat anti-ICAM2 (3C4) mAb (BioLegend, 105601), mouse anti-human pMLC2 (Ser19) mAb (Cell Signaling ,#3675), rabbit anti-human Moesin (EP1863Y) mAb (Abcam, ab52490), Alexa Fluor 488-conjugated rabbit anti-ERG2 (EPR3864) mAb (Abcam, ab196374); Secondary: Alexa Fluor 488-conjugated goat anti-rabbit IgG Ab (Thermo Fisher, A11034) , Cy3-conjucated goat anti-rabbit IgG Ab (Thermo Fisher, A10520), Cy3-conjugated goat anti-rat IgG (Thermo Fischer Scientific, A10522), Cy3-conjugated donkey anti-rabbit IgG (Thermo Fischer Scientific, A17691), Alexa Fluor 633-conjugated goat anti-rat IgG (Thermo Fisher Scientific, A21094), Alexa Fluor 633-conjugated donkey anti-goat IgG (Thermo Fisher Scientific, A21082), and Alexa Fluor 647-conjugated goat anti-rabbit IgG (Abcam, ab150135). The monoclonal antibody against murine PDGFRβ was purified from the supernatant of serum-free culture of the hybridoma cells (clone APB5) by using HiTrapTM Protein G HP column (Cytiva).

Validation

All the antibodies used in this study except for rabbit anti-GOLPH4 mAb (Abcam, ab28049) and rabbit anti-human PDGFRβ (Y92) mAb (OriGene, TA300416) are currently commercially available and validated by manufacturers as described on the following web sites or by previous reports.

rabbit anti-VE-cadherin (D87F2) mAb (Cell Signaling, 2500):
https://www.cellsignal.jp/products/primary-antibodies/ve-cadherin-d87f2-xp-rabbit-mab/2500

mouse anti-CD31 (WM59) mAb (BioLegend, 303102):
https://www.biolegend.com/ja-jp/products/purified-anti-human-cd31-antibody-883?GroupID=BLG5721

goat anti-ARPC2 pAb (Novus Biologicals, NB100-1037):
https://www.novusbio.com/products/arpc2-antibody_nb100-1037?srsltid=AfmBOooPUyifjog8Kc5_weCpk5iAPBezxatip_yqIe5xt5ozN56GUXpc

rabbit anti-TRIP10 pAb (Proteintech, 10798-1-AP):
https://www.ptglab.co.jp/products/TRIP10-Antibody-10798-1-AP.htm

goat anti-collagen type IV Ab (SouthernBiotech, 1340-01):
https://www.southernbiotech.com/goat-anti-type-iv-collagen-unlb-1340-01

rabbit anti-laminin pAb(Abcam, ab11575):

https://www.abcam.co.jp/products/primary-antibodies/laminin-antibody-ab11575.html

rabbit anti-NG2 pAb(Millipore, AB5320):
https://www.merckmillipore.com/JP/ja/product/Anti-NG2-Chondroitin-Sulfate-Proteoglycan-Antibody,MM_NF-AB5320

rat anti-mouse CD31 (MEC13.3) mAb (BD Pharmingen, 550274):
https://www.bdbiosciences.com/ja-jp/products/reagents/flow-cytometry-reagents/research-reagents/single-color-antibodies-ruo/purified-rat-anti-mouse-cd31.550274?tab=product_details

rat anti-ICAM2 (3C4) mAb (BioLegend, 105601):
https://www.biolegend.com/ja-jp/products/purified-anti-mouse-cd102-antibody-127

mouse anti-human pMLC2 (Ser19) mAb(Cell Signaling ,#3675):
https://www.cellsignal.com/products/primary-antibodies/phospho-myosin-light-chain-2-ser19-mouse-mab/3675?srsltid=AfmBOoohRFJPDCKtWPKcfLu-NdrJY90UMt1j2UQEzhndiFCHt0ERYvsr

rabbit anti-human Moesin mAb (EP1863Y) (Abcam, ab52490):
https://www.abcam.co.jp/products/primary-antibodies/moesin-antibody-ep1863y-ab52490.html

Alexa Fluor 488-conjugated rabbit anti-ERG2 (EPR3864) mAb (Abcam, ab196374):
https://www.abcam.co.jp/products/primary-antibodies/alexa-fluor-488-erg-antibody-epr3864-ab196374.html

rat anti-mouse PDGFRβ mAB (APB5):
https://www.jci.org/articles/view/15621

## Eukaryotic cell lines

Policy information about cell lines and Sex and Gender in Research

| | |
|---|---|
| Cell line source(s) | HUVECs and hLFs were purchased from Lonza. GFP-HUVECs were purchased from Anigio-Proteomie. Human pericytes from placenta (hPlPCs) were purchased from PromoCell. |
| Authentication | Authentication was conducted by each manufacturer. |
| Mycoplasma contamination | All these cell lines were tested mycoplasma-negative by each manufacturer. |
| Commonly misidentified lines (See ICLAC register) | No commonly misidentified cell lines were used in this study. |

## Animals and other research organisms

Policy information about studies involving animals; ARRIVE guidelines recommended for reporting animal research, and Sex and Gender in Research

| | |
|---|---|
| Laboratory animals | C57BL/6 WT mice (Japan SLC and CREA Japan) were used in this study. |
| Wild animals | No wild animals were used in this study. |
| Reporting on sex | Sex was not considered in this study because sex differences were not thought to have significant effect on vascular development in the mice used in the experiment, which were less than 4 days old. We used the mice at P1 to P4. |
| Field-collected samples | No field-collected samples were used in this study. |
| Ethics oversight | All experiments were carried out, in accordance with the animal care guidelines of Kumamoto University, University of Miyazaki and Nagoya City University. |

Note that full information on the approval of the study protocol must also be provided in the manuscript.

## Plants

| | |
|---|---|
| Seed stocks | *Report on the source of all seed stocks or other plant material used. If applicable, state the seed stock centre and catalogue number. If plant specimens were collected from the field, describe the collection location, date and sampling procedures.* |
| Novel plant genotypes | *Describe the methods by which all novel plant genotypes were produced. This includes those generated by transgenic approaches, gene editing, chemical/radiation-based mutagenesis and hybridization. For transgenic lines, describe the transformation method, the number of independent lines analyzed and the generation upon which experiments were performed. For gene-edited lines, describe the editor used, the endogenous sequence targeted for editing, the targeting guide RNA sequence (if applicable) and how the editor was applied.* |
| Authentication | *Describe any authentication procedures for each seed stock used or novel genotype generated. Describe any experiments used to assess the effect of a mutation and, where applicable, how potential secondary effects (e.g. second site T-DNA insertions, mosiacism, off-target gene editing) were examined.* |
